# MOF-encapsulated copper-doped carbon dots nanozymes with excellent biological activity promote diabetes wound healing

**DOI:** 10.1093/rb/rbae119

**Published:** 2024-09-30

**Authors:** Sheng Dai, Lang Jiang, Luying Liu, Zhaogui Su, Li Yao, Ping Yang, Nan Huang

**Affiliations:** Institute of Biomedical Engineering, College of Medicine, Southwest Jiaotong University, Chengdu, Sichuan 610031, China; Key Laboratory of Advanced Technologies of Materials Ministry of Education, School of Materials Science and Engineering, Southwest Jiaotong University, Chengdu, Sichuan 610031, China; Air Force Medical Center, PLA, Beijing 100074, China; Institute of Biomedical Engineering, College of Medicine, Southwest Jiaotong University, Chengdu, Sichuan 610031, China; Key Laboratory of Advanced Technologies of Materials Ministry of Education, School of Materials Science and Engineering, Southwest Jiaotong University, Chengdu, Sichuan 610031, China; Shandong Provincial Engineering Research Center of Novel Pharmaceutical Excipients and Controlled Release Preparations, College of Medicine and Nursing, Dezhou University, Dezhou 253023, China; Institute of Biomedical Engineering, College of Medicine, Southwest Jiaotong University, Chengdu, Sichuan 610031, China; Key Laboratory of Advanced Technologies of Materials Ministry of Education, School of Materials Science and Engineering, Southwest Jiaotong University, Chengdu, Sichuan 610031, China; Institute of Biomedical Engineering, College of Medicine, Southwest Jiaotong University, Chengdu, Sichuan 610031, China; Key Laboratory of Advanced Technologies of Materials Ministry of Education, School of Materials Science and Engineering, Southwest Jiaotong University, Chengdu, Sichuan 610031, China; Institute of Biomedical Engineering, College of Medicine, Southwest Jiaotong University, Chengdu, Sichuan 610031, China; Institute of Biomedical Engineering, College of Medicine, Southwest Jiaotong University, Chengdu, Sichuan 610031, China

**Keywords:** diabetic wounds, single-atom nanozyme, antioxidant activity, antibacterial activity, nano-catalytic platform

## Abstract

Poor wound healing in diabetics is primarily caused by persistently high levels of inflammation and recurrent bacterial infections. The catalytic therapy technique based on nanozyme medicine has emerged as a beacon of hope for patients with diabetic wounds. However, the use of a single-atom nanozyme may still have limitations, including nanozyme burst release, immunological clearance and insufficient antibacterial activity. To address the aforementioned problems, we provide a new nano-catalytic therapeutic agent for diabetic skin ulcers that incorporates a single-atom nanozyme with high antioxidant activity into a metal–organic framework (ZIF-Cu/C-dots). First, a Cu single-atom nanozyme supported by ultra-small carbon dots (Cu/C-dots) with high antioxidant activity was created. A nanozyme-integrated metal–organic framework was then created, utilizing Cu/C-dots as ligands and Zn^2+^ as the core metal. Cu/C-dots have good oxidase-like activity, shielding the biological system from ROS damage and reducing the expression of TNF-α and IL-1β. Zn^2+^ also has good antibacterial activity (the antibacterial rate was more than 90%). This integrated technique prevents nanozyme aggregation, improves nanozyme biocompatibility, slows down the breakdown of ZIF and allows for the regulated release of Cu/C-dots and Zn^2+^ as needed. Finally, *in vivo* studies have shown that ZIF-Cu/C-dots can effectively alleviate inflammation at the site of diabetic wounds, accelerate vascular regeneration, promote collagen deposition and enhance tissue remodeling, serving as a novel nano-catalytic platform for the treatment of wounds that are difficult to heal.

## Graphical abstract

**Figure:**
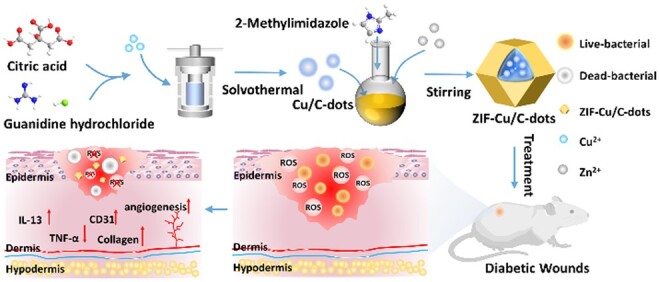

## Introduction

Diabetes is a common disease at present, about 463 million people worldwide are affected by diabetes [1]. The complications of diabetes are diverse and costly, including diabetic skin ulcers, osteoporosis and bone defects induced by diabetes, and cardiovascular diseases due to atherosclerosis in diabetic patients, posing significant threats to the health and safety of those afflicted [2]. Among these complications, diabetic skin ulcers are particularly prevalent, endangering around 25% of diabetic patients annually, and currently, effective treatments are scarce [3, 4]. The local pathological microenvironment of diabetic skin ulcers exhibits notable characteristics, such as persistent chronic inflammation, with high levels of reactive oxygen species (ROS) [5, 6] and prolonged bacterial infections, with the hyperglycemic conditions of diabetes providing a conducive breeding ground for bacteria [7–9].

Consequently, targeting these key points by regulating ROS in the local micro-environment to balance inflammatory responses while combining antimicrobial strategies is crucial for the diabetic wound’s treatment.

The emergence of ROS imbalance has been recognized as a critical mediator in the pathogenesis of inflammation [10, 11]. Antioxidant therapy utilizing antioxidant enzymes [superoxide dismutase (SOD), catalase (CAT) and glutathione peroxidase (GPx)] is considered a viable technique for treating many inflammatory diseases by reducing excess ROS [12, 13].

Natural enzymes are excellent catalysts for ROS removal in biological applications due to their high catalytic efficiency and remarkable substrate specificity. However, natural enzymes' clinical utility is limited by their potential antigenicity, poor bioavailability and low stability under pathological conditions [14]. Artificial nanozymes with enzyme-like ability, for instance polymer nanoparticles, metal nanoparticles, metal oxides, layered double hydroxides, carbon nanomaterials and so on, have increasingly come as steady and inexpensive succedaneum for natural enzymes [15–18]. Single-atom nanozymes (SAzymes) with atomically distributed metal cores have been shown to have the strongest inherent enzyme-like activity [19, 20]. SAzyme's physicochemical features, including high surface free energy, strong metal–carrier interactions and an unsaturated metal coordination environment, may improve enzyme-like activity [21]. Carbon materials are commonly employed as effective carriers of single atom catalysts because of their characteristic construction and constitutionally physical and chemical abilities. Carbon quantum dots (C-dots) appear to be the best carrier for loading metal monatomic catalysts due to their small size, strong biocompatibility, great water dispersion and high stability [22–24]. Furthermore, the nano-enzyme activity of the C-dots has steadily piqued researchers' interest. C-dots have catalytic activity similar to enzymes such as SOD, CAT and others due to their dimensional effect and abundance of active sites [25, 26]. Lin Yuehe *et al*. [27] developed and synthesized single-atom iron-doped carbon dots. Monoatomic Fe enhances the oxidase simulation ability, which can catalyze TMB oxidation with a fast response and great affinity. As a result, combining SAzyme and C-dots can not only significantly improve C-dots' catalytic performance but also provide SAzyme with numerous functions, resulting in a win–win situation.

However, there may be some limits to the use of single nano-enzymes in diabetic skin ulcers: nano-enzymes are often released quickly and are easily removed from local inflammatory regions by immune responses [28]. Hence, there is an impending need to exploit a ROS clearance nano-platform released on demand to extend the retention time of nano-enzymes in diabetic skin ulcers and adapt to the multi-functional treatment of diabetic skin ulcers. Encapsulating nano-enzymes in metal–organic frameworks (MOFs) is a promising strategy for developing complicated nano-enzymes [28]. ZIF-8, a hypotoxicity and biocompatible (MOF) made up of zinc ions and organic ligands, is stable in the physiological environment but degrades in acidic environments (including the inflammatory site) [29]. At the same time, the characteristics of nanoparticles (NPs) encapsulated in ZIF-8 can be modified to prevent NPs from aggregating [30, 31], making it ideal for developing a pH-responsive medicine release system. Furthermore, because of the biological effects of the core metal Zn ion, ZIF-8 and its matrix composites have been shown to have effective sterilizing properties [29]. Zinc ions are an efficient antibacterial agent that causes bacteria to break. The destruction of the bacterial walls leads to cytoplasm leaking and creating an alkalescent milieu, these will inhibit bacterial grow or kill them [32–34].

It is worth noting that the surface of C-dots is abundant in oxygen-containing groups, which can be combined with metal ions to form porous skeleton compounds with a stable structure. Based on the aforementioned factors, our study first designed and constructed a single-atomic Cu nanoenzyme (Cu/C-dots) supported by carbon dots with super antioxidant characteristics, and then combined it with ZIF-8 to form ZIF-Cu/C-dots nanocomposites. The integration strategy was designed to improve the stability of Cu/C-dots, achieve controlled release and improve bioavailability. In the meantime, Zn^2+^ can be set free when the decomposition of ZIF-Cu/C-dots, which has a strong antibacterial impact. Furthermore, Zn^2+^ may act synergistically with Cu/C-dots to facilitate diabetic wound healing. The chemical characterization of ZIF-8 integrated with nano-enzymes proves that they successfully load Cu/C-dots and show a typical ZIF crystal structure. Enzyme activity analysis reveals that ZIF-Cu/C-dots exhibit excellent cascade catalytic performance, including SOD simulation activity, CAT simulation activity, ·OH clearance and DPPH removal ability, making it an excellent antioxidant nano-enzyme for protecting biological systems against ROS-induced damage. Antibacterial tests demonstrate that ZIF-Cu/C-dots have outstanding antibacterial abilities to *Escherichia coli* and *Staphylococcus aureus*. *In vivo* investigations confirmed that ZIF-Cu/C-dots were effective in treating diabetes model rats' skin wounds. In short, the ZIF-Cu/C-dots nanocomposites we proposed are antibacterial, capable of removing various ROS on demand, anti-inflammatory and so on. In a special skin wound environment, which may be an attractive treatment for chronic wounds of diabetes.

## Materials and methods

### Materials

Citric acid, guanidine hydrochloride, copper chloride (CuCl_2_), sodium hydroxide standard solution, 2-methylimidazole, zinc acetate (C_4_H_6_O_4_Zn), diphenyl-2-diphenyl-1-(2,4,6-trinitrobenzene) hydroxyl group (DPPH), methanol solution, methylene blue powder and ferric chloride (FeCl_2_) are purchased from the Shanghai Aladdin Reagent network. Sigma-Aldrich Corporation (USA) supplied the following materials: neotetrazolium blue chloride (NBT), xanthine (X), xanthine oxidase (OX), lipopolysaccharide (LPS), 2′,7′-dichlorodihydrofluorescein diacetate (DCFH-DA), acridine orange (AO) and iopropyl (PI). We obtain LB AGAR and LB broth from Guangdong Huankai Microbial Technology Co., Ltd., China. We purchase streptozocin (STZ) from Solarbio Technologies Ltd.

### Cu/C-dot synthesis and characterization

Mixing citric acid (1 g) and guanidine hydrochloride (2 g) with 20 ml RO water, and then 20 mg of CuCl_2_ was added. The pH was changed to 11 by using NaOH. Then the solution was put into a high-pressure vessel after reacting for 6 h at 160°C. Filtering out the large particles when the mixture has cooled to room temperature. To get rid of the unreacted molecules, a dialysis bag (500 D) was performed after this. Finally, freeze-drying was used to produce Cu/C dots. Field emission transmission electron microscopy (TEM, JSM-2100F, JEOL, Japan) was used to observe the morphology of Cu/C-dots. Using a spherically aberration-corrected transmission electron microscope (AC-STEM, JEM-ARM200F), the distribution of single-atom Cu was examined. The elemental composition was tested by using X-ray photoelectron spectroscopy (XPS; K-Alpha, ThermoElectron, USA).

### Preparation of ZIF-Cu/C-dots

Mix 500 μl 2-methylimidazole (125 mg/ml) and Cu/C-dots for 5 min. Then add 500 μl zinc acetate (21 mg/ml) after stirring for 5 min. The solution was centrifuged, washed and samples were collected. According to the concentration of Cu/C-dots, the samples were successively named ZIF, ZIF-Cu/C-dots-1, ZIF-Cu/C-dots-2, ZIF-Cu/C-dots-3, ZIF-Cu/C-dots-4 and ZIF-Cu/C-dots-5.

The morphological characteristics of ZIF-Cu/C-dots were tested by using field emission scanning electron microscopy (SEM, JSM-7800F, JEOL, Japan). The distribution of elements was analyzed by an energy dispersive spectrometer (EDS). Using XPS to test the elemental composition of ZIF-Cu/C-dots.

### Responsive release

ZIF-Cu/C-dots were prepared into a 10 mg/ml solution with PBS (pH 5.4, 6.8, 7.4), carefully sealed into a dialysis bag (1000 Da), then put it in 20 ml corresponding PBS, and finally placed in a constant temperature shaking table. The solution was collected at 2, 4, 6, 12, 24 and 48 h, respectively, for the absorbance test.

### DPPH scavenging

DPPH, 0.04 mg/ml, was mixed with the sample in the dark for 5 min. The absorbance test was then performed at 517 nm.

### Enzyme assay similar to SOD

In order to create the superoxide anion (·$O_{2}^{-}$), X (0.09 mg/ml) and XO (0.05 U/ml) were mixed together. Following a 5-min reaction in which various samples were added in proportion, NBT (1.6 mg/ml) was added, and after 5 min of color development, absorbance was tested at 550 nm.

To further identify the removal of ·$O_{2}^{-}$ from ZIF-Cu/C-dots-2, EPR was employed.

### Scavenging of hydroxyl radicals (·OH)

About 10 μl H_2_O_2_ (35%, Bioss Biotechnology Co., Ltd., Beijing, China) and 10 μl FeCl_2_ (8 mg/ml) were mixed to create ·OH. Following a 5-min reaction, samples were introduced. After continuous reaction for 5 min, MB solution was added. Following a 15-min reaction, the UV absorption spectra were examined.

### Assay for catalase (CAT)-like enzymes

The CAT-like ability of ZIF-Cu/C-dots was tested by two-component TMB (Bioss) as color-developing substrate. About 700 μl PBS (pH 6.8) was mixed with 100 μl ZIF-Cu/C-dots (5 mg/ml) solution, 100 μl H_2_O_2_ (35%), 300 μl TMB A solution and 300 μl TMB B solution. After reacting for 6 h, detecting by enzyme-labeled apparatus.

To further identify the removal of ·OH from ZIF-Cu/C-dots-2, EPR was employed.

### Antibacterial activity test

The antibacterial activity of ZIF-Cu/C-dots was evaluated with *S. aureus* and *E. coli*. First, adjusting the bacterial density to 1 × 10^8^ CFU/ml with normal saline. Then, mixing bacterial suspension with ZIF-Cu/C-dots (20 μg/ml), cultured for 24 h. Dilute the bacteria by an order of magnitude after dispersing them evenly. About 50 μl bacterial suspension was placed on a sterile AGAR plate and evenly dispersed. After incubation for 1 day, photographs were taken, colonies were counted and bacterial clearance was calculated.

Put a sterile silicon wafer in 24-well plate, followed by bacterial (1 × 10^8^ CFU/ml). After incubation at 37°C for 12 h, ZIF-Cu/C-dots were added, and after continued culture for 24 h, the silicon wafer with bacteria was removed, cleaned with NaCl, fixed with glutaraldehyde for 24 h. After three rounds of NaCl cleaning, gradient dehydration with ethanol and drying, the bacteria were coated with gold using an ion sputtering device and then observed under a SEM.

*E. coli* and *S. aureus* were mixed with samples (bacteria: 1 × 10^7^ CFU/ml, a ZIF-Cu/C-dots-2: 20 μg/ml), added 500 μl mixed solution to the 48-well plate, incubated for 2 days. Gently washed off with NaCl, the biofilm was stained with crystal violet and photographed. Finally, the biofilm was washed with an alcohol solution, and quantitative analysis was carried out by the enzyme-labeled method.

### *In vitro* blood and cell compatibility

After anesthetizing SD rats, blood was collected using a negative pressure tube with anticoagulants. A 5 ml NaCl solution was mixed with 4 ml whole blood. Then, adding 9.7 ml NaCl solution and 100 μl ZIF-Cu/C-dots (10 mg/mL) to 200 μl above mixture blood. After incubation for 1 h, centrifuge at 3000 r/min for 5 min and finally take 150 μl solution at 540 nm for absorbance test.

Human umbilical artery vascular endothelial cells (HUVECs) were co-cultured with different concentrations of ZIF-Cu/C-dots to evaluate cytotoxicity. Mixing 10 μl ZIF-Cu/C-dots (1 mg/ml) with 490 μl serum medium with cells (2 × 10^5^ cells/ml). After 3 days of culture at 37°C, the cell compatibility was tested with AO/PI live/dead and CCK-8 (Biosharp Technology Ltd., Anhui, China) cell activity assay kits.

### Intracellular antioxidant test

Macrophages were purchased from Servicebio in Wuhan China. MAs was first cultured, and then LPS (1 μg/ml) was added one day later, the culture was continued for 48 h, and then ZIF-Cu/C-dots-2 (20 μg/ml) was added. Cultured for one day, intracellular ROS were observed by dichlorofluorescein (DCFH-DA) fuel (dichlorofluorescein is a fluorescent probe for detecting ROS). Finally, cell imaging was performed by laser confocal microscopy to evaluate intracellular ROS levels, and finally, the release of TNF-α and IL-1β by cells was tested by ELISA kit.

### Skin wound healing in rats given a diabetes model

#### Creation of a model of diabetic rats

Every animal experiment followed the guidelines authorized by Southwest Jiaotong University's Animal Care and Use Committee [SWJTU-2403-NSFC (116)].

As model animals, male Sprague Dawley rats (SD, 300–350g) were employed. After 12 h of continuous water fasting, they were given a peritoneal injection of streptozotocin (STZ, 60 mg/kg). Fasting blood glucose was randomly measured three times in 5 days, and if the blood glucose was higher than 11.1 mmol/L, the diabetes model was considered successful. If blood sugar does not rise to 11.1 mmol/L, repeat the procedure until the diabetes model is established.

#### Repairing wounds using ZIF-Cu/C dots

After anesthetizing diabetic mice, a full-layer skin wound with a diameter of 1 cm was formed on the back of the rats using a sterile hole punch. On the incision, apply 100 μl of ZIF-Cu/C-dots-2 drops (20 μg/ml). Each group had wound photos taken 0, 5, 9 and 14 days apart, those pictures were used to measure wound healing.

#### Immunohistochemical and histological evaluation

At days 3, 7 and 14, the skin tissue was carefully collected, cleaned with NaCl for three times and fixed with paraformaldehyde for preservation. Subsequently, paraffin embedding and tissue section were performed, and H&E, Masson and immunohistochemical staining (CD31, TNF-α) were further performed to observe skin regeneration, collagen deposition, neovascularization and inflammatory response.

### Statistics

Graphpad Prism and Origin Pro were used to analyze the data, which was then displayed as mean ± standard deviation. The Students' test was used to determine differences between the two groups, while the one-way ANOVA test was utilized to compare the two. A *P* value of less than 0.05 (*P < 0.05, **P < 0.01, ***P < 0.001) indicates statistical significance.

## Results and discussion

### Formation and characterization

**Figure 1.**
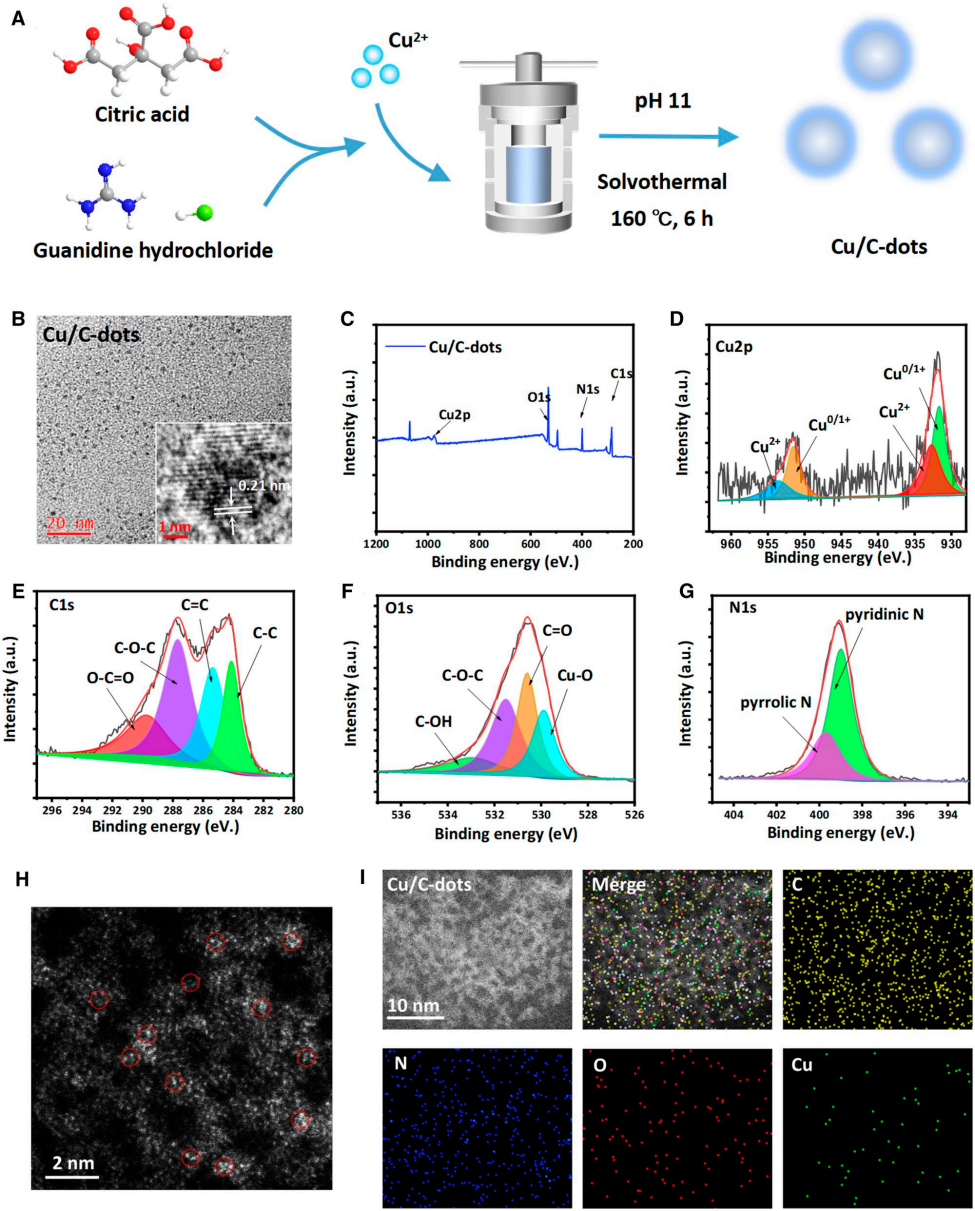
(**A**) Schematic illustration of the formation of Cu/C-dots. (**B**) TEM and HRTEM image. The full XPS survey spectra (**C**) and high-resolution XPS spectra of Cu2p (**D**), C1s (**E**), O1s (**F**) and N1s (**G**) of Cu/C-dots. (**H**) HAADF-STEM image of Cu/C-dots. (**I**) Elemental mapping.

Firstly, Cu/C-dots were produced utilizing a one-step hydrothermal technique with small molecules of citric acid and guanidine hydrochloride as carbon sources and Cu as a doping metal (Figure 1A). The TEM and HRTEM results (Figure 1B) revealed that Cu/C-dots were monodisperse spherical particles. The size is less than 5 nm, and the lattice spacing is 0.21 nm, which corresponds to the graphite-carbon (1 0 0) layer spacing [35]. XPS data (Figure 1C) revealed that Cu/C-dots were primarily made up of C, N, O and Cu elements. The Cu element appeared in the form of Cu^0/1+^ or Cu^2+^ (Figure 1D), which served as the structural foundation for enzymatic processes. Cu/C-dots were made of pyridinic and pyrrolic N structures, as well as hydrophilic functional groups such as –C–OH, –COOH and so on, according to high-resolution C, N and O data (Figure 1 E–G). These hydrophilic groups provided them with good stability and biocompatibility. Simultaneously, these groups promote metal–ion coordination. AC-STEM was used to demonstrate the presence of single copper atom, as demonstrated in the HAADF-STEM image (Figure 1H), Cu single atom may be identified as isolated brilliant spots equally dispersed across the carbon carrier. The material's composition was further evaluated using energy dispersive X-ray spectroscopy (EDS) and elemental mapping. The elemental map (Figure 1I) revealed that C, N, O and Cu were uniformly distributed throughout the nanostructure.

**Figure 2.**
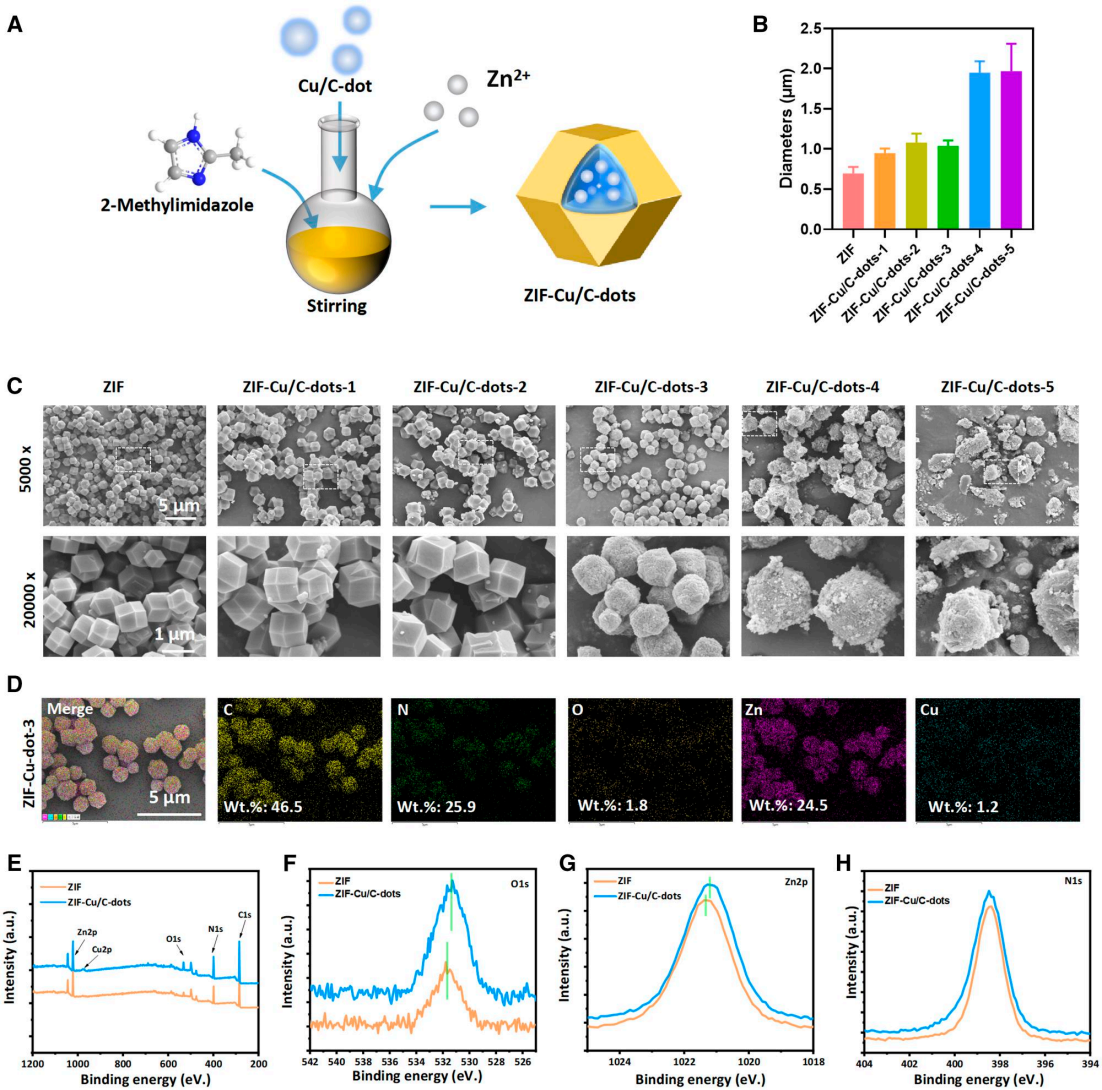
(**A**) The preparation process diagram of ZIF-Cu/C-dots. (**B**) Particle size statistics. (**C**) SEM images of ZIF and ZIF-Cu/C-dots-1-5. (**D**) EDS element mapping of ZIF and ZIF-Cu/C-dots. (**E**) The full XPS survey spectra. The high-resolution XPS spectra of O1s (**F**), Zn2p (**G**) and N1s (**H**).

Then, a metal–organic framework (ZIF-Cu/C-dots) was built using Cu/C-dots as a ligand and Zn^2+^ as the core metal (Figure 2A). Cu/C-dots were distributed in 2-methylimidazole solutions and quickly added to a zinc acetate solution. Cu/C-dots are integrated into the composite through competitive coordination with Zn^2+^. The combination became muddy quickly after adding Zn^2+^, whereas the nucleation process in the solution without Cu/C-dots (pure ZIF-8) was slower (see Supplementary Video Attachment). The surface of Cu/C-dots contains several functional groups, such as hydroxyl and carboxyl, which are negatively charged under synthetic circumstances and have a high affinity for Zn^2+^ and can promote the nucleation of ZIF-8. The solution was milky white following ZIF-8 synthesis, but it gradually changed to yellow after Cu/C-dot adding (Supplementary Figure S1). The SEM results (Figure 2C) revealed that ZIF-8 had a regular 12-hedral structure, a smooth surface and a particle size of approximately 695 nm. The surface roughness of ZIF-Cu/C-dot particles increased when the Cu/C-dots increased, as did the particle size (Figure 2B). However, it was important to note that excessive Cu/C-dot addition causes the size of ZIF-Cu/C-dots to rise unnaturally, making it difficult to create regular nanoparticles. Furthermore, EDS data (Figure 2D) revealed that the nanoparticles exhibited clear elemental mapping of C, N, O, Cu and Zn. This demonstrated the successful insertion of Cu/C-dots.

The XPS data (Figure 2E–H) showed that the obvious signals of C, N, O, Cu and Zn in the composite material. Furthermore, the high-resolution element spectra indicated that the binding energy of Zn2p was gradually transferred from 1021.38 to 1021.18 eV when Cu/C-dots exists, the binding energy of O1s was gradually transferred from 531.78 to 531.28 eV, and the binding energy of N1s was not shifted, indicating the formation of a new coordination bond between Zn and O. As a result, Cu/C-dots may be proven to have played an important part in ZIF-8’s formation. Therefore, it is more than just a physical enclosure; it is a chemical cooperation with ZIF-8.

### Responsive release of ZIF-Cu/C-dots

Diabetic skin wounds are acidic due to chronic inflammation and bacterial infection [36], therefore, the drug release of ZIF-Cu/C-dots was evaluated at different pH values. As a result, ZIF-Cu/C-dots were found to release Cu/C-dots in a responsive manner. According to the release within 48 h (Supplementary Figure S2A), the release of Cu/C-dots followed pH5.4 > pH6.8 > pH7.4 at the same time. SEM results (Supplementary Figure S2B) also demonstrated the responsiveness of ZIF-Cu/C-dots, and the introduction of Cu/C-dots could appropriately slows down the decomposition of ZIF to achieve long-term drug-controlled release.

### ROS clearance capability of ZIF-Cu/C-dots

Under normal physiological settings, the intracellular antioxidant system clears ROS, which are byproducts of normal cell metabolic processes. An imbalance between the ROS and antioxidant systems can cause oxidative stress, resulting in hazardous side effects [37]. Therefore, we examined the ROS clearance capacity of ZIF-Cu/C-dots *in vitro*.

**Figure 3.**
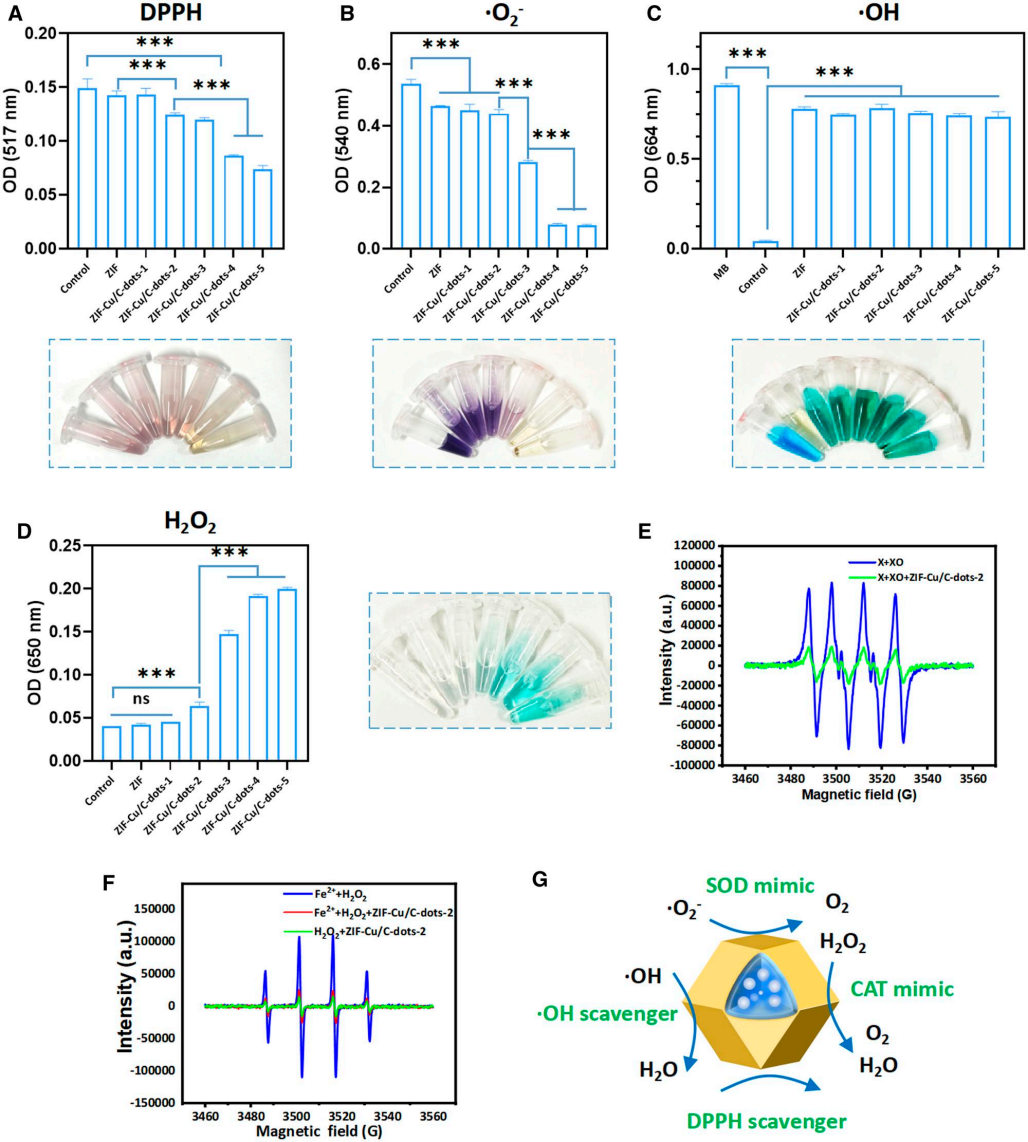
ROS clearing activities of ZIF-Cu/C-dots. (**A**) Absorption spectra of DPPH reacted with or without ZIF or ZIF-Cu/C-dots, the tubes (inset) show the color change of DPPH. (**B**) NBT's absorption spectra after reacting with ·$O_{2}^{-}$ in the presence or absence of ZIF or ZIF-Cu/C-dots. The tubes (inset) display the color change that results from this reaction. The inset display the color change of MB. (**C**) Absorption spectra of MB reacted with ·OH with or without ZIF or ZIF-Cu/C-dots. The inset display the color change of TMB responded with H_2_O_2_. (**D**) Absorption spectra of TMB reacted with H_2_O_2_ with or without ZIF or ZIF-Cu/C-dots. (**E**) EPR spectra of ·$O_{2}^{-}$. (**F**) EPR spectra of ·OH. (**G**) Schematic of antioxidative activities of ZIF-Cu/C-dots.

DPPH is a typical nitrogen-containing free radical, and removing it is one approach for determining the catalytic activity of samples. They interact with DPPH by swapping hydrogen atoms or electrons to eliminate free radicals, altering the appearance and characteristics of DPPH. As shown in Figure 3A, ZIF-Cu/C-dots demonstrated antioxidant activity proportional to Cu/C-dot concentration. The more Cu/C-dots loaded, the lighter the color of DPPH, indicating that ZIF-Cu/C-dots has a higher DPPH clearance capacity.

To further investigate the ROS elimination ability of ZIF-Cu/C-dots, the NBT method was used to identify their SOD-like activity. NBT, as a probe of •$O_{2}^{-}$, will be decreased to purple methylhydrazine, with maximum absorbance at 540 nm. The SOD enzyme can remove •$O_{2}^{-}$, which inhibits the synthesis of methylhydrazine. The OD values at 540 nm revealed that the SOD-like ability of ZIF-Cu/C-dots was positively linked with the concentration of Cu/C-dots (Figure 3B). Furthermore, it was discovered that ZIF may eliminate portions •$O_{2}^{-}$, which could be attributed to the extra impact of the Zn metal active site in ZIF.

Another type of ROS that contributes to inflammation is hydroxyl radicals (•OH), which are formed from Fe^2+^ and H_2_O_2_. Therefore, we next examined whether ZIF-Cu/C-dots could remove •OH. Methylene blue (MB) has an absorption peak at 664 nm and can be faded by •OH oxidation caused by Fenton reagent, making it useful as a •OH indicator. As seen in Figure 3C, the individual MB solution appears blue and absorbs significantly. When Fenton's agent reacts with MB, it immediately faded with little absorption at 664 nm, but this was prevented when ZIF and ZIF-Cu/C-dots were added to the system. This finding indicates that ZIF and ZIF-Cu/C-dots can remove •OH.

The POD-like activity of ZIF-Cu/C-dots was investigated by TMB, a commonly used POD substrate. In the presence of ZIF-Cu/C-dots, the combined TMB and H_2_O_2_ solution turned a distinct blue color (Figure 3D), revealing an evident OX-TMB characteristic absorption peak (650 nm). Simultaneously, the characteristic absorption peak intensity of OX-TMB increased with the increase of Cu/C-dots concentration, revealing that Cu/C-dots showed obvious POD-like activity.

The EPR experiments (Figure 3E and F) further proved that ZIF-Cu/C-dots can remove ·$O_{2}^{-}$ and ·OH. And ZIF-Cu/C-dots will not react with H_2_O_2_ to produce ·OH.

Taken together, these findings indicated that ZIF-Cu/C-dots can clear a variety of ROS (Figure 3G), which has potential in the treatment of inflammatory illnesses.

### Antibacterial properties of ZIF-Cu/C-dots

**Figure 4.**
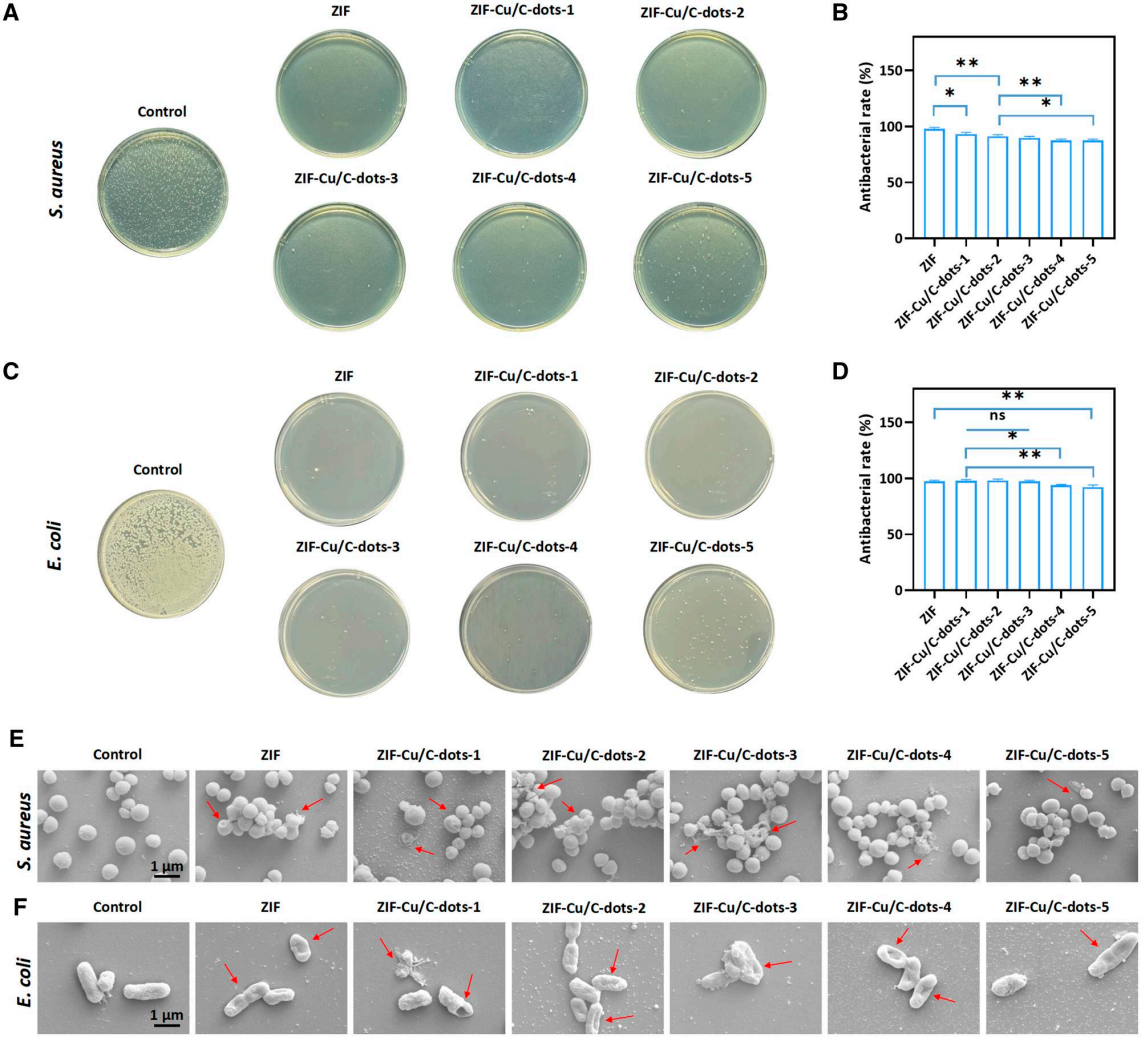
Antibacterial activities of ZIF-Cu/C-dots. (**A**) Typical photo of *S. aureus* colonies. (**B**) Antimicrobial rate statistics: *S. aureus*. (**C**) Typical photo of *E. coli* colonies. (**D**) Antimicrobial rate statistics: *E. coli*. (**E**) Typical SEM pictures of *S. aureus* and (**F**) *E. coli* after different treatments.

The high sugar environment of diabetes wounds allows bacteria to thrive, which is one of the primary reasons diabetic wounds heal. To assess the antibacterial activity of ZIF-Cu/C-dots, we used *S. aureus* and *E. coli* as typical bacteria in diabetic wounds (Figure 4A and D). The bacteriostatic rates of ZIF and ZIF-Cu/C-dots-1-5 against *S. ureus* were 97.97%, 93.26%, 91.32%, 89.79%, 87.36% and 87.43%, respectively, and against *E. coli* were 97.33%, 97.77%, 98.13%, 97.43%, 94.03% and 92.17%. The antibacterial activity of ZIF and ZIF-based composites is based on zinc ions, and the interaction of Cu/C-dots with Zn^2+^ may impede the release of Zn^2+^, affecting the antibacterial performance of ZIF-Cu/C-dots.

The integrity and morphological alterations of the bacterial membrane were examined using SEM. As shown in Figure 4E and F, bacterial in the untreated control group had normal morphology with smooth surfaces and intact cell membranes, whereas bacteria after ZIF and ZIF-Cu/C-dots-1-5 treatment had significant changes, with bacterial membranes showing serious wrinkles and damage, resulting in cell content leakage. SEM results revealed that ZIF and ZIF-Cu/C-dots-1-5 successfully killed bacteria by disrupting their membranes.

**Figure 5.**
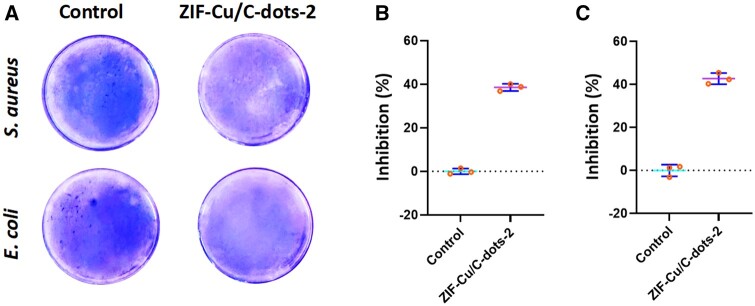
(**A**) Crystal violet staining picture of *S. aureus* and *E. coli* biofilms treated with ZIF-Cu/C-dots-2. Inhibition percentage: (**B**) *S. aureus* and (**C**) *E. coli* biofilms formation.

Since biofilm formation often leads to increased bacterial resistance to antimicrobials, the antibiofilm activity of ZIF-Cu/C-dots-2 was also evaluated (Figure 5A–C). Crystal violet staining showed that ZIF-Cu/C-dots-2 inhibited the formation of *S. aureus* and *E. coli* biofilms. The inhibition rates of *S. aureus* and *E. coli* biofilm formation were 38.53% and 42.6%, respectively.

### Biocompatibility of ZIF-Cu/C-dots

**Figure 6.**
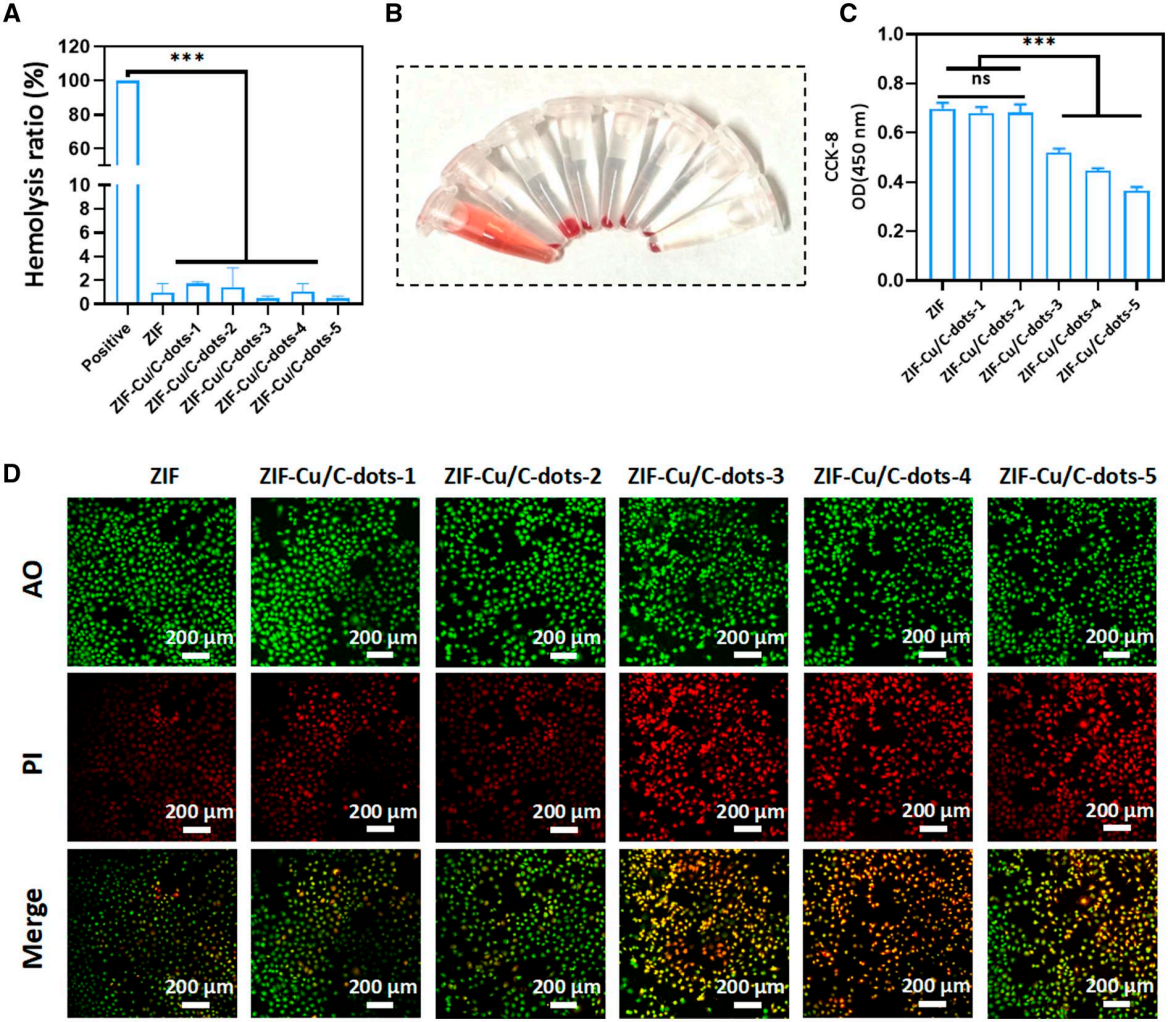
Blood and cell compatibility of ZIF-Cu/C-dots. (**A**) Hemolysis ratio of ZIF or ZIF-Cu/C-dots. (**B**) Macro-graphs of hemolysis. (**C**) CCK-8 of ECs cultured with ZIF or ZIF-Cu/C-dots. (**D**) Fluorescent images of ECs (green, AO; red, PI).

Good biocompatibility is required for the use of biomaterials. We tested the biosafety of ZIF-Cu/C-dots *in vitro* using the hemolysis assay, cell viability kit CCK-8 test and cell viability (AO)/death (PI) staining. As shown in Figure 6A and B, the supernatant of all samples except the positive control was translucent, and normal blood cells were deposited at the bottom of the centrifuge tube. After calculation, the hemolysis rate of all samples was less than 5%, indicating good blood safety. ZIF, ZIF-Cu/C-dots-1 and ZIF-Cu/C-dots-2 (20 μg/ml) showed no difference in the absorbance of ECs cells co-cultured in CCK-8 for 3 days (Figure 6C). Indicating that ZIF, ZIF-Cu/C-dots-1 and ZIF-Cu/C-dots-2 had good cytocompatibility. In the ZIF-Cu/C-dots-3-5 group, CCK-8 absorbance fell considerably, indicating some cytotoxicity. This was further supported by live/dead staining data (Figure 6D).

ZIF-Cu/C-dots-2 (20 μg/ml) is an optimal alternative for future investigations due to its catalytic, antibacterial and biocompatibility capabilities.

### Intracellular antioxidant and anti-inflammatory properties

**Figure 7.**
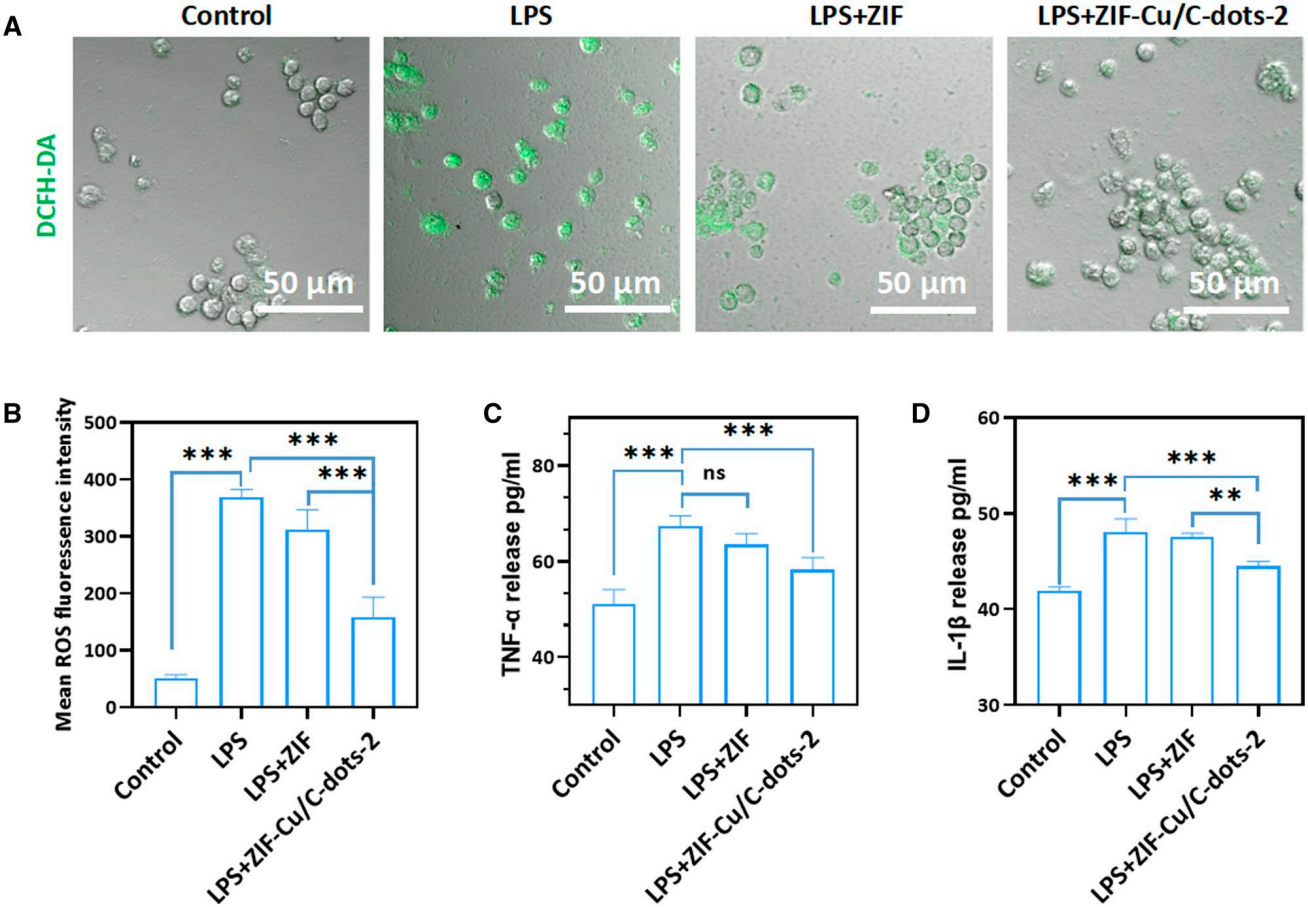
Intracellular antioxidant activities of ZIF-Cu/C-dots. (**A**) The CLSM pictures for intracellular ROS detection using the DCFH-DA assay after various treatments. (**B**) Mean ROS fluorescence intensity of the DCFH-DA assay upon different treatment. (**C**) Relative inflammatory factor expression of TNF-α in the MAs upon different treatment. (**D**) Relative inflammatory factor expression of IL-1β in the MAs upon different treatment.

Excessive ROS accumulation causes oxidative stress in surrounding tissues, which leads to increased inflammation [38]. Given the remarkable ROS clearance ability of ZIF-Cu/C-dots, we investigated their intracellular antioxidant capability. LPS was chosen as a stimulus to cause oxidative stress in macrophages, and the ROS fluorescent probe DCFH-DA was employed to assess its antioxidant activity. DCFH-DA was oxidized to DCFH when ROS exists, which exhibited green fluorescence. As demonstrated in Figure 7A and B, the LPS-treat group showed strong fluorescence expression, showing that LPS may stimulate ROS formation, but the fluorescence intensity of the LPS + ZIF group dropped slightly, but not significantly. The fluorescence intensity of the LPS + ZIF-Cu/C-dots-2 group was dramatically lowered, demonstrating that ZIF-Cu/C-dots-2 could remove intracellular ROS and hence protect against endogenous oxidative stress. Excessive production of pro-inflammatory factors (TNF-α and IL-1β), during inflammation can cause tissue damage. We assessed ZIF-Cu/C-dots-2's anti-inflammatory effects by measuring TNF-α and IL-1β levels in an LPS-induced inflammation model. LPS treatment considerably elevated TNF-α and IL-1β levels compared to normal MAs cells (Figure 7C and D), demonstrating a successful *in vitro* inflammatory model. It is clear that co-incubation with Cu/C-dots-2 reduces cytokine production considerably. Those results revealed that ZIF-Cu/C-dots-2 will effectively alleviate inflammation *in vitro* through ROS clearance and reduction of pro-inflammatory factors.

### ZIF-Cu/C-dots improves diabetic wound healing

**Figure 8.**
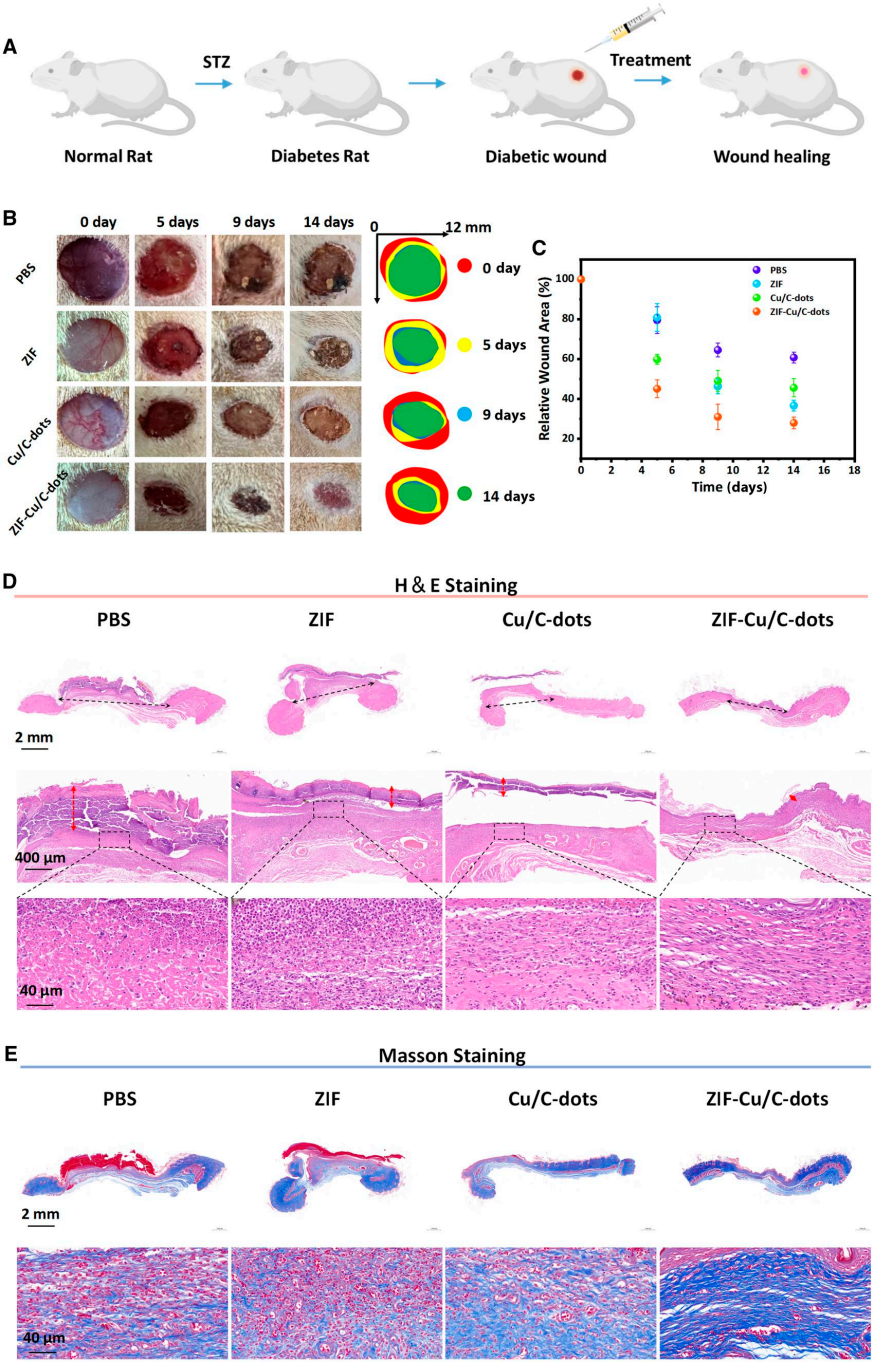
Treatment of ZIF-Cu/C-dots for in diabetic wound healing. (**A**) The program of treatment process. (**B**) Typical wound photos of wounds taken at various stages. (**C**) Relative wound area statistics at different time after surgery. (**D**) H&E staining in each group on Day 14. (**E**) Masson staining in each group were performed on Day 14.

Streptozotocin (STZ) was injected into SD rats to create a diabetic model (Figure 8A). The rats' blood glucose was tested at random over a three-day period, and the effectiveness of diabetic model-building was success when the blood glucose level exceeded 11.1 mmol/l. The model animals were then randomly allocated into four groups: PBS, ZIF, Cu/C-dots and ZIF-Cu/C-dots. In Figure 8B, the ZIF-Cu/C-dots group healed faster than the other groups, with only 23.9% of the original wound remaining after 14 days (Figure 8C). Histological staining was used to assess wound tissue regeneration across treatment groups. H&E staining photos (Figure 8D) revealed that thick scab tissue still existed in the PBS, ZIF and Cu/C-dots groups. The difference was that the ZIF-Cu/C-dots group had a nearly complete epidermal layer. In addition, neutrophils rose considerably in the PBS and ZIF groups, indicating a strong inflammatory response. In contrast, the Cu/C-dots and ZIF-Cu/C-dots treated groups had lower inflammatory cell infiltration and inflammation, with the ZIF-Cu/C-dots treated group having more regular cell organization. Masson staining was employed to further assess collagen fiber production (Figure 8E). In comparison to the other groups, ZIF-Cu/C-dots treatment significantly increased collagen deposition. The overall findings indicate that ZIF-Cu/C-dots can effectively heal chronic wounds.

**Figure 9.**
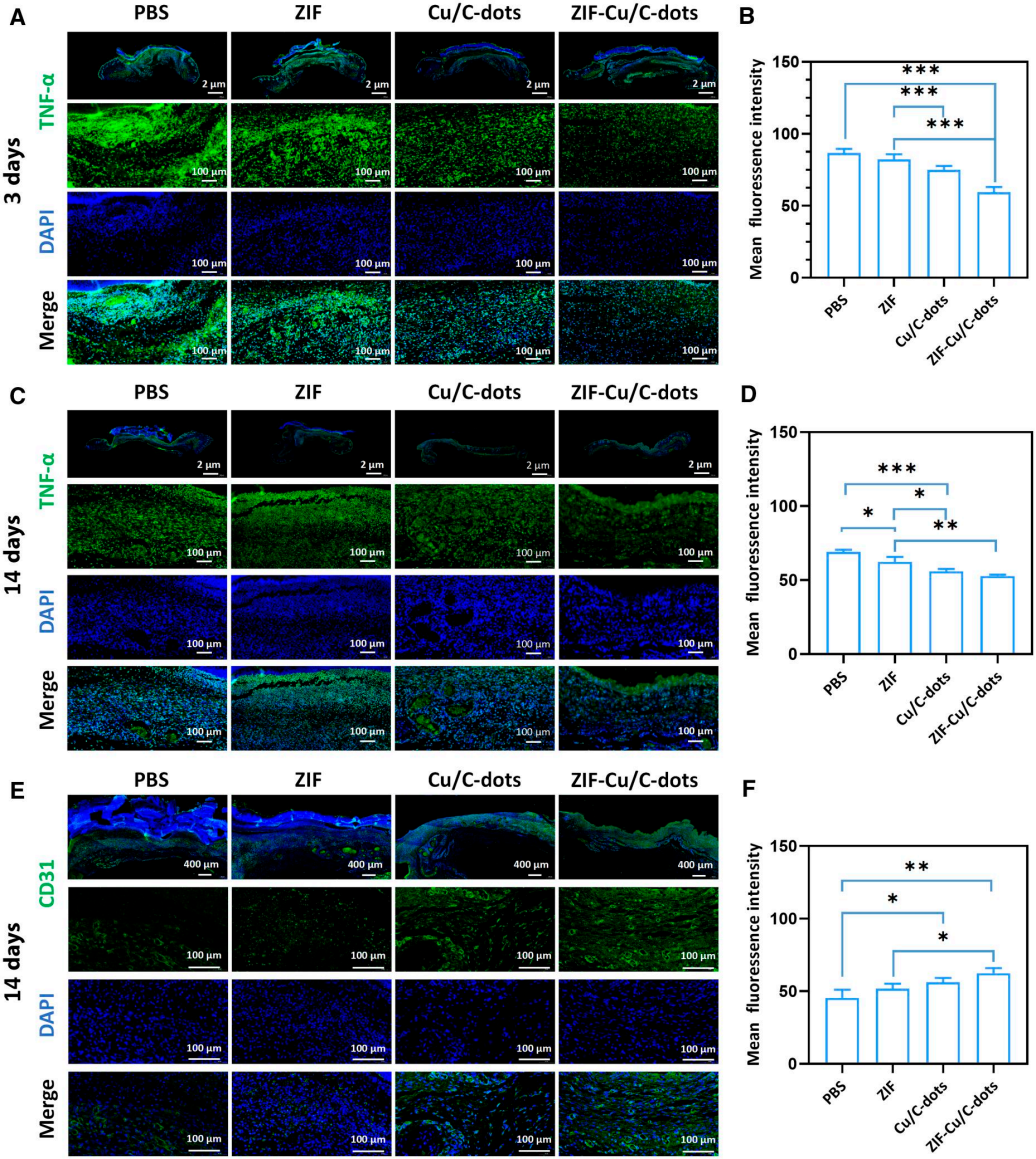
TNF-α staining (**A**) and mean fluorescence intensity (**B**) in wound sections on day 3. TNF-α staining (**C**) and mean fluorescence intensity (**D**) in wound sections on day 14. CD31 staining (**E**) and mean fluorescence intensity (**F**) in wound sections on day 14.

Immunofluorescence labeling was used to measure TNF-α and CD31 levels in wound tissue. It was allowed to compare the impact of various treatment options on inflammation and angiogenesis. TNF-α staining results are shown in Figure 9A–D. Conversely, in contrast to the control group, the expression of TNF-α in wound tissue treated with Cu/C-dots and ZIF-Cu/C-dots was significantly reduced on Days 3 and 14. The above results indicated the Cu/C-dots and ZIF-Cu/C-dots-treatment groups could decease the early inflammation, importantly, the ZIF-Cu/C-dots group showed better treatment outcomes, which could be attributed to ZIF's long-term therapeutic effect by limiting the rapid release of Cu/C-dots. CD31 is an endothelial cell marker in angiogenesis. Those findings (Figure 9E and F) revealed that the ZIF-Cu/C-dots group had a higher intensity of green fluorescence (indicating CD31) than the other three treatments. Taken together, these data indicate that ZIF-Cu/C-dots can efficiently lower inflammatory factor expression, boost angiogenesis and increase collagen deposition, thereby expediting wound healing.

## Conclusion

Constant inflammation and repeated bacterial infections severely hinder the healing process of damaged tissue; by embedding Cu/C-dots with multiple enzyme activities into the shell of ZIF-8, we successfully created the nanozyme-integrated ZIF-8 composite material ZIF-Cu/C-dots. This complex nano enzyme showed strong cascade SOD and CAT mimic activity, as well as effective elimination of ·OH and DPPH, indicating promise for reducing intracellular ROS and inflammation. Furthermore, ZIF-Cu/C-dots-2 has excellent antibacterial and biocompatibility properties. *In vitro* anti-bacterial tests indicated that the antibacterial rates of ZIF-Cu/C-dots-2 against *S. aureus* and *E. coli* were 91.2% and 98.1%, the biofilms’ inhibition rates were 38.53% and 42.6%, respectively. *In vitro* inflammatory cell model experiments showed that ZIF-Cu/C-dots-2 treatment is expected to reduce intracellular ROS and relieve inflammation. *In vivo* tests have revealed ZIF-Cu/C-dots-2 will significantly reduce any inflammatory response at the diabetic wound site, accelerate vascular regeneration, collagen deposition and promote wound healing. Overall, the multifunctional complex nano-enzyme synergistic therapeutic strategy developed in this study based on multiple enzyme-like activities has great potential for treating the poor wound healing in diabetics.

## Supplementary Material

rbae119_Supplementary_Data
