# Supplementary material for: MOF-encapsulated copper-doped carbon dots nanozymes with excellent biological activity promote diabetes wound healing
Source: Regen Biomater. 2024 Sep 30;11:rbae119. doi: 10.1093/rb/rbae119 (PMC11580684; doi:10.1093/rb/rbae119)
Supplement: rbae119_Supplementary_Data [file rbae119_supplementary_data.zip › Supplementary Materials.docx]

**Supplementary Materials**

**MOF-Encapsulated Copper-Doped Carbon Dots Nanozymes with Excellent Biological Activity Promote Diabetes Wound Healing**

Sheng Dai^1, 2 #^, Lang Jiang^3 #^, Luying Liu^1, 2, 4 *^, Zhaogui Su^1, 2^, Li Yao^1, 2^, Ping Yang^1 *^, and Nan Huang^1^

1. Institute of Biomedical Engineering, College of Medicine, Southwest Jiaotong University, Chengdu 610031, Sichuan, China.

2. Key Laboratory of Advanced Technologies of Materials Ministry of Education, School of Materials Science and Engineering, Southwest Jiaotong University, Chengdu 610031, Sichuan, China.

3. Air Force Medical Center, PLA, Beijing 100074, China.

4. Shandong Provincial Engineering Research Center of Novel Pharmaceutical Excipients and Controlled Release Preparations, College of Medicine and Nursing, Dezhou University, Dezhou 253023, China.

# These authors contributed equally to this work.

* Correspondence should be addressed to Ping Yang; yangping8@263.net; Luying Liu; 19827558031@163.com


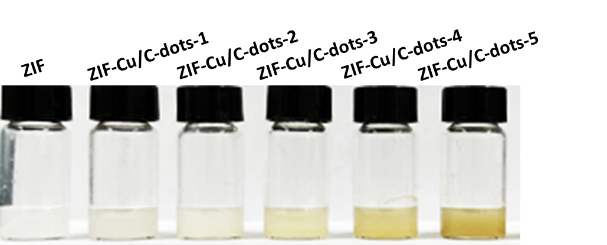


**Figure S1.** Cell phone photo of ZIF-Cu/C-dots


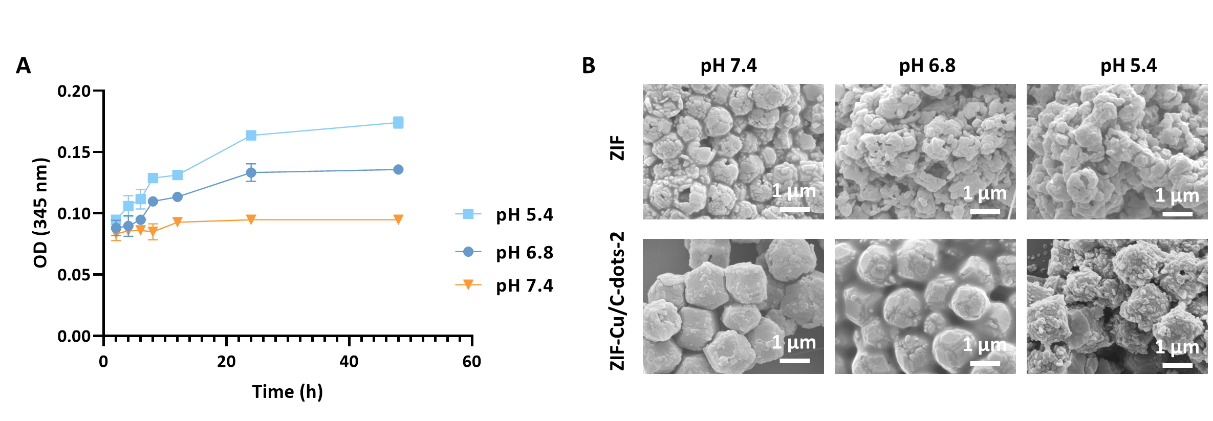


**Figure S2.** (A)Cu/C-dots release curve at different pH. (B) SEM images of ZIF-Cu/C-dots soaked at different pH for 24 h.

**Figure S3.** Standard release curve of TNF-α.

**Figure S4.** Standard release curve of IL-1β.
